# Anti-Photoaging Effect of Soluble Microneedles Loaded with Hydroxytyrosol

**DOI:** 10.3390/ijms27021005

**Published:** 2026-01-20

**Authors:** Jie Wang, Gaofei Zhu, Mengke Han, Xinyu Hou, Yishu Wang, Xiuhua Zhang, Jinhua Zhang, Huarong Shao, Fei Liu

**Affiliations:** 1 School of Pharmacy, Shandong University of Traditional Chinese Medicine, Jinan 250355, China; 2 Engineering Research Center for Sugar and Sugar Complex, National-Local Joint Engineering Laboratory of Polysaccharide Drugs, Key Laboratory of Carbohydrate and Glycoconjugate Drugs, Shandong Academy of Pharmaceutical Science, Jinan 250101, China; 3 Key Laboratory of Brain, Cognition and Education Sciences, Ministry of Education, Institute for Brain Research and Rehabilitation, South China Normal University, Guangzhou 510631, China

**Keywords:** skin photoaging, ultraviolet radiation, hydroxytyrosol, soluble microneedles, antioxidant

## Abstract

Skin photoaging, marked by structural and functional changes, is mainly caused by long-term ultraviolet (UV) exposure. This study sought to create hydroxytyrosol (HT)-loaded soluble microneedles (HT MNs) and thoroughly assess their anti-photoaging effects and underlying mechanisms in vitro and in vivo. The optimized HT MNs, featuring tips with 10% HT + 5% hyaluronic acid (HA) and a backing layer of 10% polyvinyl pyrrolidone (PVP), demonstrated robust mechanical strength (withstanding an axial force of 10 N without fracture), adequate penetration depth (>200 μm), and efficient skin self-recovery post-removal. In vitro, HT MNs notably boosted cell viability, reduced reactive oxygen species (ROS) levels, and suppressed senescence-associated β-galactosidase (A-β-Gal) expression in UVA-exposed human skin fibroblasts (HSF). In vivo, in a UVA + UVB-irradiated mouse model, HT MNs significantly enhanced skin hydration and elasticity, increased collagen density (confirmed by Masson staining), decreased malondialdehyde (MDA) content, and elevated the activities of glutathione (GSH), catalase (CAT), and glutathione peroxidase (GSH-Px). Western blot analysis further revealed that HT MNs upregulated the expression of collagen type I alpha 1 (COL1A1), elastin (ELN), hyaluronan synthase 2 (HAS2), and filaggrin (FLG), while downregulating matrix metalloproteinase 1. Overall, these findings suggest that HT MNs effectively mitigate UV-induced photoaging through antioxidant, anti-senescence, and extracellular matrix (ECM)-regulating mechanisms, underscoring their potential as a novel transdermal anti-photoaging therapy.

## 1. Introduction

Skin photoaging, characterized by wrinkles, sagging, and pigmentation, is caused by accumulated ultraviolet (UV)-induced damage to the extracellular matrix (ECM) [1]. According to statistics, photoaging accounts for about 80% of skin aging [2,3]. UV is divided into Ultraviolet A (UVA) (320–400 nm) and Ultraviolet B (UVB) (280–320 nm). UVA has strong penetration ability and can penetrate the epidermis directly to the dermis layer. UVB has short waves and only about 5–10% can reach the surface and can be absorbed by the epidermis and part of the dermis [4]. Excessive UVA irradiation of skin cells can lead to a large accumulation of ROS in the dermis cells, which in turn triggers an inflammatory cascade reaction, such as the inflammatory pathway induced photoaging mediated by nuclear factor kappa-B (NF-κB) and tumor necrosis factor alpha (TNF-α) [5,6]. Meanwhile, reactive oxygen species (ROS) play a crucial role in regulating collagen metabolism, leading to increased expression of matrix metalloproteinases (MMPs) and accelerating skin aging [7,8,9]. ROS causes damage to DNA, proteins, and lipids in skin cells, leading to metabolic disorders, oxidative stress, inflammatory reactions, cell apoptosis, degradation of ECM such as collagen, dehydration, wrinkles, sagging, and loss of elasticity, and even increasing the risk of skin cancer [10,11,12].

With the continuous growth of the economy, people’s awareness of health and skin care is gradually increasing, especially their attention to photoaging issues [13]. Photoaging not only affects individuals’ appearance and mental health, but also brings huge economic burdens to individuals and society [14,15,16]. Therefore, studying photoaging and its preventive measures has important social and economic significance [17,18]. At present, although traditional prevention and treatment methods such as sunscreen are widely used, they lack sufficient protection for deep skin layers [19]. The traditional treatment methods for photoaging include laser power therapy and the application of antioxidants such as retinoids and vitamin E, but they have problems such as high cost, short efficacy, and irritating side effects. Therefore, developing efficient, safe, and highly targeted anti photoaging strategies has become a research hotspot [20]. With the increasing emphasis on biosafety, more and more studies have revealed the important role of plant derived natural products in treating UV radiation-induced skin photoaging [21]. Natural products have the advantages of photoprotection, antioxidant, and low-risk properties, which can effectively prevent skin photodamage and skin cancer caused by UV radiation, as well as reduce irritation, allergies, phototoxic reactions, photosensitivity, and the production of reactive oxygen species [22,23,24].

The photoprotective ability of polyphenols in natural products is particularly noteworthy [25,26,27,28,29]. The potential utility of polyphenols is assumed in the pathological environment of skin response to UV radiation, including inflammatory cascade reactions, oxidative disturbances, and DNA damage [30,31,32]. Hydroxytyrosol (HT) is a polyphenolic compound that naturally exists in olive oil and is the main source of fat in the Mediterranean diet. It has attracted much attention due to its strong antioxidant, anti-inflammatory, and anti apoptotic properties [32,33,34,35,36,37]. However, traditional transdermal drug delivery systems have problems such as low transdermal absorption efficiency and easy drug degradation, which limit the widespread application of HT in clinical practice [38].

The specification of microneedles (MNs) is generally 10–2000 μm in height and 10–50 μm in diameter [39]. It generally cause temporary physical damage to the stratum corneum of the skin through micron-sized needle tips, creating microporous channels through which drugs are directly transported to the epidermis or upper dermis [39]. MN technology has demonstrated significant safety advantages and low adverse reaction rates in the field of subcutaneous injection [40]. The MN technology of local drug delivery system can quickly and effectively penetrate the stratum corneum barrier of the skin, achieving local and targeted drug delivery to the dermis layer, thereby significantly increasing local drug concentration and greatly reducing systemic side effects. Soluble MNs represent an innovative minimally invasive transdermal drug delivery technology [41]. They utilize a micron-scale needle-like array made from water-soluble or biodegradable materials to temporarily create microchannels in the outermost stratum corneum layer of the skin. With the characteristics of precision, painlessness, self-degradation, and high compliance, soluble microneedles are emerging as a revolutionary tool in the field of transdermal drug delivery [42,43]. This innovative technology provides a new solution to overcome the bottleneck problem of traditional transdermal drug delivery [44].

The aim of this study is to develop soluble MNs loaded with HT and evaluate their anti photoaging efficacy through in vitro cell models and in vivo animal experiments. The research focuses on the regulatory effects of HT MNs on UV induced oxidative stress, collagen metabolism disorders, and skin structure damage, and explores their molecular mechanisms, in order to provide a novel delivery system and theoretical basis for the prevention and treatment of photoaging.

## 2. Results

### 2.1. Screening of Materials and Fabrication Parameters for MN Arrays

Experimental results demonstrate that MNs (Sample No. 1) prepared with deionized water as the solvent exhibit superior performance in formability, mechanical strength, and bubble volume control. In summary, the optimal MN fabrication parameters were identified as 10% (*w*/*v*) PVP for the backing layer and a 15 min vacuum treatment (Parameter Set No. 1). This combination preserved needle tip integrity while enhancing substrate toughness. For the drying step, constant-temperature incubator drying at 37 °C was selected as the optimal method, balancing drying efficiency with MN molding quality (Table S1).

**Figure 1:**
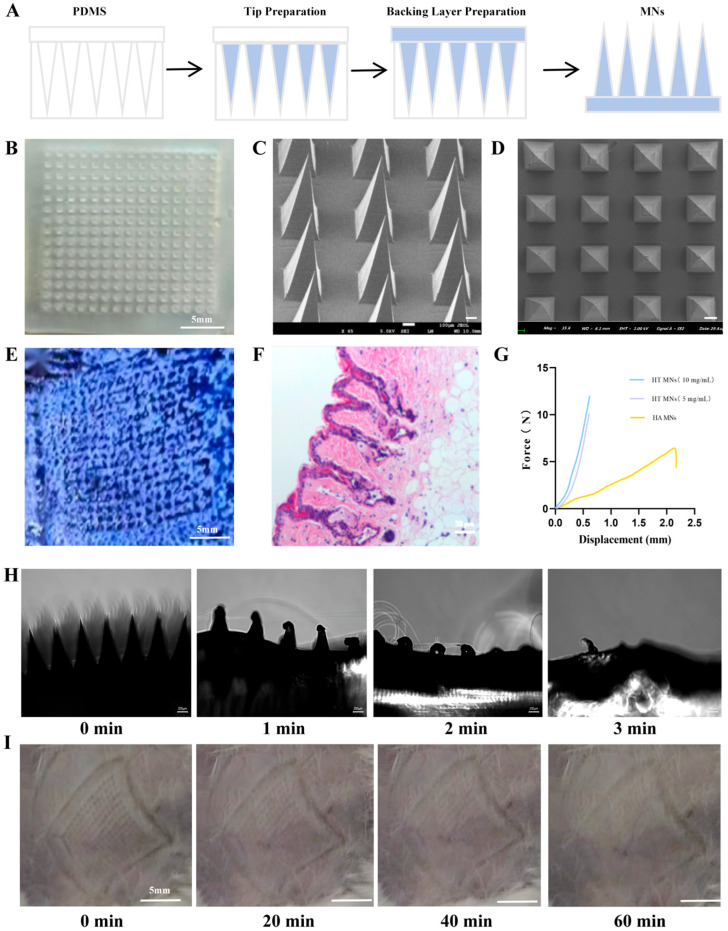
Preparation and characterization of MNs patches. (**A**) A stepwise schematic diagram outlines the preparation process of HA-based MN patches. (**B**) Macroscopic image of the fabricated MN patch captured under a camera. Scale bar = 5 mm. (**C**) High-resolution SEM image displaying the lateral profile of MN structures. Scale bar = 100 μm. (**D**) SEM image showing the planar arrangement of MN tips. Scale bar = 400 μm. (**E**) Fluorescence microscopy image of MN-treated cells stained with TB to assess membrane integrity. Scale bar = 5 mm. (**F**) Histological section of mouse skin tissue post-MN implantation, stained with H&E to evaluate tissue response. Scale bar = 100 μm. (**G**) Quantitative assessment of the average axial stress applied per needle in the MN patch (n = 3). Data are presented as mean ± standard deviation (SD). (**H**) Sequential images depicting the indentation of an HA MN tip into the skin over time. Scale bar = 100 μm. (**I**) Time-lapse images illustrating skin recovery after MN patch removal. Scale bar = 5 mm.

As a major component of the ECM, HA maintains body water balance and intracellular homeostasis. Due to its biocompatibility and mechanical properties, HA is widely used as the base material for MN fabrication. In this study, commercially available polydimethylsiloxane (PDMS) was employed as the MN mold (S4). The HA-based MNs were prepared via a two-stage molding process: Tip formation: A 5% (*w*/*v*) HA solution was mixed with HT. 100 μL of the mixture was cast into the PDMS mold cavities and vacuum-degassed (−0.08 MPa, 15 min) to eliminate air bubbles. Base formation: The mold surface was subsequently filled with 150 μL of a hybrid solution containing HA (3%) and PVP (10%) to form the MN base (Figure 1A). Figure 1B shows the optical microscopy image of the fabricated MN patch, which consists of a 10 × 10 MN array with a needle height of 600 μm and inter-needle spacing of 400 μm (Figure S2).

### 2.2. Morphological and Structural Characterization of MN Arrays

Through scanning electron microscopy (SEM) observation of multiple batches of MN arrays, the following geometric parameters were quantified: needle tip sharpness angle of 15° ± 1.2°, base diameter of 344.4 ± 8.7 μm, inter-tip spacing of 621.5 ± 12.4 μm, and average needle height of 548.5 ± 9.3 μm. These dimensions confirm sufficient mechanical strength to penetrate the stratum corneum and deliver drugs into the dermal layer. Morphological analysis revealed smooth needle surfaces free of visible defects, with uniform tip geometry and ordered array distribution. The overall formability was excellent, characterized by high needle density, minimal porosity (<2%), and flat substrate bases (Figure 1C,D).

### 2.3. Analysis of Mechanical Characteristics

Mechanical compression tests conducted using a universal testing machine revealed that high-concentration HT MN arrays sustained an axial load of 12.0 ± 1.1 N at 0.6 mm displacement without structural failure, while low-concentration HT MNs withstood 10.2 ± 0.9 N under identical conditions. In contrast, pure HA MNs fractured at 4.1 ± 0.3 N when displaced by 2.0 mm (Figure 1E). Statistical analysis (two-tailed *t*-test, *p* < 0.01) confirmed significantly superior mechanical strength of HT MNs compared to HA MNs (n = 5 arrays per group).

### 2.4. Transdermal Puncture Efficacy and Safety Profile in Skin Recovery

The TB staining experiment demonstrated that the puncture efficiency of HT MNs was 90 ± 5% (Figure 1E). Hematoxylin eosin (H&E) staining results revealed that the insertion depth of the MNs reached the dermis layer, meeting the design requirements. These data indicated that the fabricated MNs possessed high hardness and good toughness, were resistant to bending and fragmentation, and could effectively penetrate the stratum corneum of the skin. Immediately after removing the MNs 10 s post-puncture, fine channels with a diameter of approximately 50 ± 5 µm were visible on the skin surface. The edges of these channels were well-defined, and there was no obvious redness, swelling, or bleeding. After 5 min, the channels began to gradually narrow, with some already starting to close, and the diameter reduced to 30 ± 3 µm. The edges of the channels became blurred, and the surrounding skin exhibited slight contraction. After 10 min, the diameter further decreased to 10 ± 2 µm, and most of the channels were nearly closed, leaving only minor depressions. After 15 min, the channels on the skin surface were completely closed, with no visible puncture marks to the naked eye, and only slight differences in epidermal smoothness could be detected through high-magnification microscopic observation. The complete closure of the channels through the skin’s natural repair mechanism, without inducing inflammation or scarring, demonstrated the high reversibility of the skin damage caused by the MNs, meeting the biosafety requirements for clinical applications (Figure 1H). Meanwhile, microscopic examination of MN patches at different puncture times revealed that the needle tips were completely dissolved within 3 min (Figure 1I).

### 2.5. Quantitative Analysis of Therapeutic Payload in HT MN Arrays

The test sample, prepared by dissolving the HT MN tip array (n = 3) in acetonitrile/water (50:50, *v*/*v*), was subjected to HPLC analysis (λ = 254 nm). The chromatogram exhibited a single characteristic peak with identical retention time to the HT reference standard (Figure S1), confirming compound identity. Quantification was performed using an external standard calibration curve in the range of 1–100 μg/mL. The mean total drug loading of the MN array was determined as 62.15 ± 0.87 mg (n = 3), corresponding to a recovery rate of 103.6 ± 1.5% relative to the theoretical design value (60 mg). The coefficient of variation (CV%) for inter-tip drug distribution was calculated as 2.3%, indicating high encapsulation efficiency (>95%) and uniform spatial distribution across the array. A standardized dose of 60 mg was selected for subsequent in vitro release studies (Figure S3).

### 2.6. Establishment of UV-Induced HSFs Damage Model and HT MNs’ Protective Efficacy Assessment

**Figure 2:**
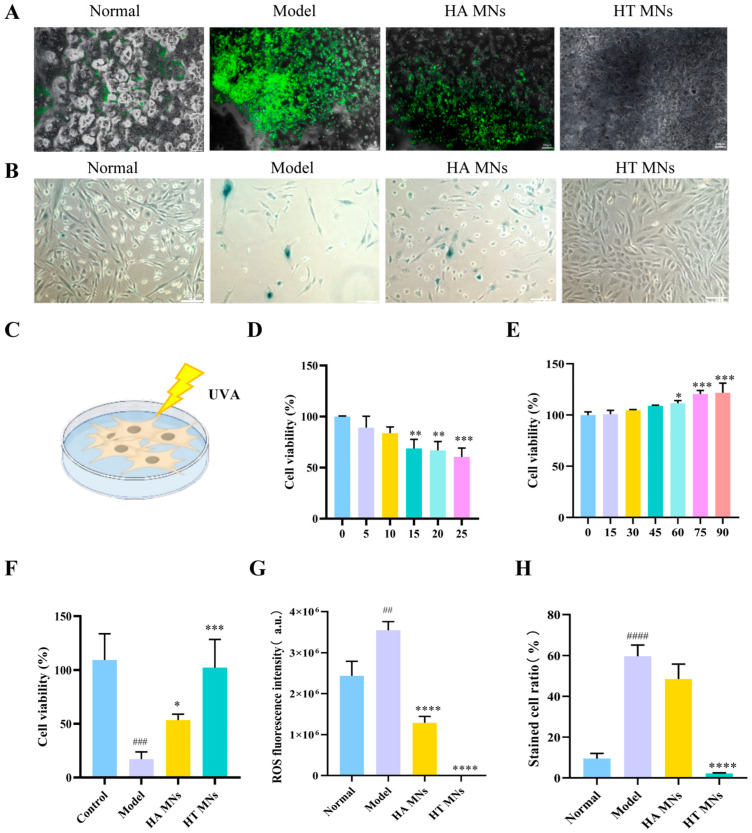
Evaluation of anti-aging effects of HT MNs in a UVA-induced dermal fibroblast model. (**A**) Representative fluorescence microscopy images (scale bar = 200 μm) showing intracellular ROS production in HSFs across experimental groups following UVA irradiation (10 J/cm^2^). DCFH-DA probe was used for ROS detection, with green fluorescence intensity indicating ROS levels. (**B**) Microscopic visualization (scale bar = 200 μm) of β-Galexpression (senescence-associated marker) in HSFs from different treatment groups post-UVA exposure. Blue staining intensity correlates with cellular senescence progression. (**C**) Schematic illustration of the experimental workflow for UVA irradiation of HSFs, including pretreatment conditions and irradiation parameters. (**D**) Dose–response curve demonstrating the impact of varying UVA intensities (0–20 J/cm^2^) on HSF viability, assessed via CCK-8 assay at 24 h post-irradiation. (**E**) In vitro cytotoxicity profile of RHT MNs at different concentrations (0–100 μM) on HSFs, measured by CCK-8 assay after 24 h incubation. (**F**) Comparative analysis of HSF viability across experimental groups following UVA irradiation (10 J/cm^2^), with control groups including untreated, UVA-only, and HA MN-treated cells. (**G**) Quantitative fluorescence intensity analysis of ROS production in HSFs from different treatment groups, normalized to control. Data derived from DCFH-DA staining (as shown in panel (**A**)). (**H**) Quantification of β-galactosidase-positive cells in HSF populations across treatment groups, expressed as percentage of total cells. Statistical significance: * *p* < 0.05, ** *p* < 0.01, *** *p* < 0.001, **** *p* < 0.0001, ## *p* < 0.01, ### *p* < 0.001, #### *p* < 0.0001. (n ≥ 3, mean ± SD).

To establish an in vitro model of UV-induced skin fibroblast damage, HSFs were exposed to varying doses of UVA radiation, and cell viability was assessed using the CCK-8 assay to determine the optimal modeling dose. As illustrated in Figure 2C, a significant inverse correlation was observed between HSF survival rate and UVA radiation dose (r = −0.92, *p* < 0.001). Progressive inhibition of cell viability with increasing UVA dosage (0–50 mJ/cm^2^) was demonstrated, validating its efficacy in inducing HSF damage for constructing a reliable photoaging cell model. Quantitative analysis (Figure 2D) revealed that cell viability decreased markedly at 15 mJ/cm^2^ compared to the control group (0 mJ/cm^2^; *p* < 0.01). Based on the dose–response curve, the half-maximal inhibitory concentration (IC_50_) of UVA on HSFs was calculated as approximately 25 mJ/cm^2^. To ensure moderate and reproducible cellular damage, this dose was selected for subsequent photoaging model induction.

Prior to determining the concentration of HT MNs, their biocompatibility with HSFs was evaluated. cell counting kit-8 (CCK-8) assay results (Figure 2E) demonstrated that HT enhanced HSF viability within a specific concentration range (10–100 mg/mL), with maximal stimulation observed at 90 mg/mL (125 ± 3.2% of control; *p* < 0.001). Consequently, 90 mg/mL was chosen as the experimental concentration for HT MNs in this study.

Following successful model establishment and dose optimization, the protective efficacy of HT MNs against UVA-induced HSF injury was investigated. As shown in Figure 2F, post-25 mJ/cm^2^ UVA exposure, the model group exhibited a sharp decline in cell viability (42.3 ± 2.1%) compared to the normal control group (set at 100%; *p* < 0.001), confirming model validity. In contrast, treatment with HT MNs significantly restored cell viability to 78.6 ± 3.5% (*p* < 0.01 vs. model group). Notably, the HT MNs group demonstrated superior protection compared to the HA MN matrix control (61.2 ± 2.8%; *p* < 0.05). These findings strongly support that HT MNs effectively counteracted UVA-induced cytotoxicity in HSFs through UV-protective mechanisms mediated by its core active component, HT.

### 2.7. Influence of HT MNs on A-β-Gal Expression

A-β-Gal serves as a reliable biomarker for cellular senescence, where senescent cells exhibit characteristic blue coloration. As demonstrated in Figure 2A, the model group displayed marked morphological alterations, including a substantial increase in β-galactosidase-positive (blue-stained) cells (*p* < 0.001 vs. control), indicating accelerated cellular aging. Conversely, the HT treatment group exhibited restored cellular morphology with a significant reduction in blue-stained cells (*p* < 0.01 vs. model group), suggesting attenuated senescence. The HA control group showed morphological features comparable to the model group, with persistent elevation of blue-stained cells (*p* > 0.05 vs. model group), indicating limited anti-aging efficacy. These findings collectively demonstrate that HT MNs exert potent anti-senescent effects by mitigating UVA-induced cellular aging, likely attributable to the intrinsic anti-aging properties of HT (Figure 2G).

### 2.8. Modulatory Impact of HT MNs on Oxidative Stress Status

Excessive accumulation of ROS constitutes a primary mechanism underlying UV-induced cellular damage. To evaluate the regulatory effects of HT MNs on UVA-mediated oxidative stress, we employed the (2′,7′-dichlorodihydrofluorescein diacetate) DCFH-DA fluorescent probe to quantify intracellular ROS levels. The assay principle relies on the following sequential events: (1) non-fluorescent DCFH-DA permeates cell membranes and undergoes hydrolysis by intracellular esterases to generate DCFH, which is retained within the cytoplasm; (2) in the presence of ROS, DCFH undergoes oxidation to form highly fluorescent DCF, with fluorescence intensity directly proportional to ROS concentration.

As demonstrated in Figure 2B, UVA-irradiated model group exhibited significantly elevated green fluorescence intensity compared to the normal control group (*p* < 0.01), confirming robust ROS generation in HSFs. Conversely, HT MN intervention resulted in a marked reduction in intracellular fluorescence intensity relative to the model group (*p* < 0.0001), indicating potent ROS scavenging activity. Notably, the antioxidant efficacy of HT MNs significantly surpassed that of HA MNs containing only the matrix component (*p* < 0.001). These findings collectively demonstrate that HT MNs possess superior antioxidant capacity by effectively eliminating UVA-induced ROS overproduction, thereby mitigating oxidative stress injury. This effect primarily stems from the intrinsic antioxidant properties of HT, the core active constituent of HT MNs.

### 2.9. Comprehensive Apparent Assessment of the Photostability and Anti-Aging Performance of HT MNs

**Figure 3:**
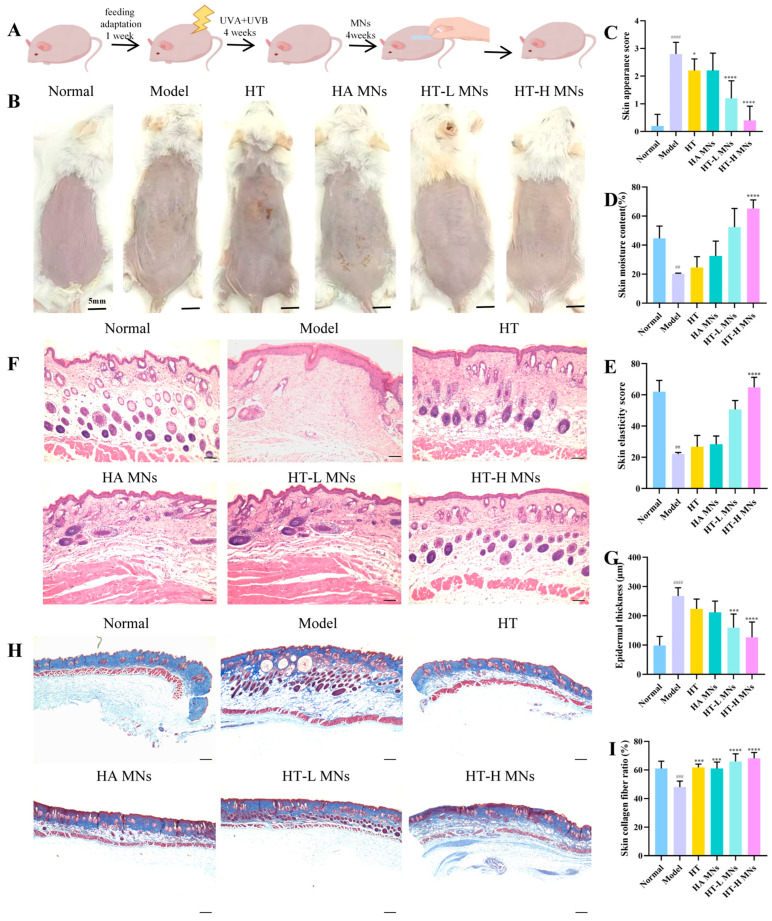
In vivo anti-photoaging efficacy of HT MNs in a UV-induced murine model. (**A**) Schematic representation of the photoaging induction protocol (chronic UVA/UVB irradiation, 365/290 nm LED, 5 mW/cm^2^ each, 30 min/day for 28 days) and experimental design for anti-aging intervention. (**B**) Macroscopic observation of skin wrinkles and damage in each treatment group post-intervention. (**C**) Quantitative macroscopic scoring of skin conditions based on wrinkle severity, laxity, and roughness (0–4 point scale). (**D**) Measurement of stratum corneum hydration. (**E**) Assessment of skin elasticity. (**F**) H&E staining of mouse dorsal skin (scale bar = 50 μm), showing epidermal thickness and dermal structure. (**G**) Quantification of epidermal thickness (μm) through histological analysis (n = 3/group). (**H**) Masson’s trichrome staining of mouse skin (scale bar = 50 μm), highlighting collagen fiber distribution. (**I**) Analysis of collagen content in skin (expressed as mean ± SD, n = 3). Statistical significance: * *p* < 0.05, *** *p* < 0.001, **** *p* < 0.0001, ## *p* < 0.01, ### *p* < 0.001, #### *p* < 0.0001.

To further validate the in vivo anti-photoaging efficacy of HT MNs, we established a murine skin photoaging model induced by chronic UVA + UVB irradiation. The therapeutic outcomes were quantitatively evaluated using a macroscopic scoring system (Figure 3A), which assessed four key parameters: wrinkle severity, skin laxity, roughness, and erythema (each graded 0–4 points). As demonstrated in the scoring results (Figure 3B,C), mice in the normal control group exhibited smooth, taut skin with no visible wrinkles (score = 0). In contrast, the model group developed pronounced static wrinkles, epidermal laxity, and coarse texture following periodic UVA/UVB exposure, achieving a significantly higher mean score than all other groups (*p* < 0.0001), confirming successful model establishment. In the treatment cohorts, the hydroxytyrosol low-dose (HT-L) MNs group displayed primarily slight dynamic wrinkles, with a score significantly lower than the model group (*p* < 0.0001), suggesting partial mitigation of photoaging damage. The hydroxytyrosol high-dose (HT-H) MNs group demonstrated skin conditions comparable to the normal control, with scores not only significantly lower than the model group (*p* < 0.0001) but also superior to the HT-L MNs group (*p* < 0.05), indicating stronger wrinkle-inhibitory effects.

Using the Skin Moisture Tester (RBX-916, Real Bubee, Jinan, China), the skin moisture content and skin elasticity of mice were evaluated. As can be seen from Figure 3D,E. In Figure 3D, which depicts the skin moisture content (%), the normal group exhibits the highest moisture level. The model group shows a significant decrease in skin moisture compared to the normal group. The HT group and various MN-related treatment groups (such as HA MNs, HT-L MNs, HT-H MNs) demonstrate different degrees of improvement in skin moisture content compared to the model group, with the HT-H-MN group showing the most prominent increase, reaching a level close to that of the normal group and showing a significant difference (**** indicates *p* < 0.0001). In Figure 3E, which represents the skin elasticity score, a similar trend is observed. The normal group has the highest skin elasticity score, while the model group has the lowest. The treatment groups show varying degrees of enhancement in skin elasticity compared to the model group, and the HT-H-MN group again shows the most significant improvement, with a substantial difference from other groups (**** indicates *p* < 0.0001).

### 2.10. Comprehensive Histopathological Examination of Photoaged Skin Post-Treatment Modalities

To evaluate the impact of different treatments on the tissue architecture of photoaged skin, skin samples from each group underwent H&E staining and Masson’s trichrome staining. The normal group exhibited intact skin architecture with a clear epidermal-dermal junction, densely packed and regularly arranged dermal collagen fibers, orderly cellular distribution, and minimal inflammatory cell infiltration (Figure 3F). In contrast, the model control group demonstrated typical photoaging pathology: significant epidermal hyperplasia with uneven thickness, disorganized and sparse dermal collagen fibers accompanied by fiber fragmentation and reduction, cellular disarray, and pronounced inflammatory cell infiltration. The HA MNs group showed partial improvement compared to the model group, with a clearer epidermal-dermal boundary and denser collagen fiber arrangement, though residual fiber breaks and inflammatory infiltration persisted. The HT-L MNs group exhibited marked structural enhancement, including reduced epidermal hyperplasia, more compact and organized collagen fibers, and significantly diminished fiber fragmentation and inflammation. The HT-H MNs group demonstrated the most pronounced recovery, with histomorphology closely resembling the normal control group: uniform epidermal thickness, a well-defined epidermal-dermal junction, closely packed and regular collagen fibers with minimal fractures, and negligible inflammatory infiltration. Notably, the model and HA MNs groups displayed highly similar H&E staining patterns, indicating severe structural damage and limited repair efficacy of pure HA MN matrices in photoaging. To elucidate the delivery mechanism and matrix contribution, we included traditional topical HT application and HA MN groups as controls. The standalone HT cream group showed limited improvement, with scores statistically indistinguishable from the model group (*p* > 0.05) but significantly inferior to the HT-H MNs group (*p* < 0.0001). Similarly, the HA MN group (matrix-only control) exhibited no significant anti-aging effects (all *p* > 0.05 vs. model group; *p* < 0.0001 vs. HT-H MNs group) (Figure 3G).

Masson’s trichrome staining, which specifically stains collagen fibers blue, directly visualized collagen morphology, distribution, and integrity. The normal group showed dark blue, densely packed, and regularly arranged collagen fibers without degeneration or fractures. In contrast, the model group exhibited significantly lighter staining, reflecting reduced collagen content, loose and disorganized fiber arrangement, and extensive degeneration/fractures. The HA MNs group demonstrated deeper collagen staining and closer fiber arrangement compared to the model group, though degeneration persisted. The HT-L MNs group showed further enhanced collagen staining intensity and orderliness, with reduced degeneration/fractures. The HT-H MNs group most closely resembled the normal control group in collagen staining depth and arrangement, indicating near-normal collagen synthesis and tissue repair (Figure 3H,I).

The histopathological findings from H&E and Masson’s trichrome staining strongly correlated with macroscopic skin appearance scores. These results collectively confirm that the HT MN system effectively repairs UV-induced skin photoaging damage, likely through mechanisms involving collagen fiber regeneration/remodeling, inflammatory suppression, and restoration of normal skin architecture. Additionally, a dose-dependent effect was observed, with the HT-H MNs group showing superior therapeutic efficacy, while the HA MNs group lacked significant advantages, underscoring the critical role of the active ingredient HT in treatment outcomes.

### 2.11. HT MNs Outperform HT in Enhancing Skin Antioxidant Defense and ECM Repair in Photoaging

To further elucidate the impact of different treatments on the skin antioxidant system, we measured MDA content, GSH levels, and activities of key antioxidant enzymes CAT; GSH-Px in skin tissues from each group. The results revealed no significant difference in skin appearance scores between the HT group and the model control group. Although the HT group exhibited a trend toward improvement in biochemical indicators, the reductions in MDA content, increases in GSH levels, and enhancements in CAT and GSH-Px activities were not statistically significant, with effects far inferior to those of the HT MNs group. These findings suggest that traditional transdermal delivery of HT may be insufficient to effectively improve the overall antioxidant capacity of skin tissues.

**Figure 4:**
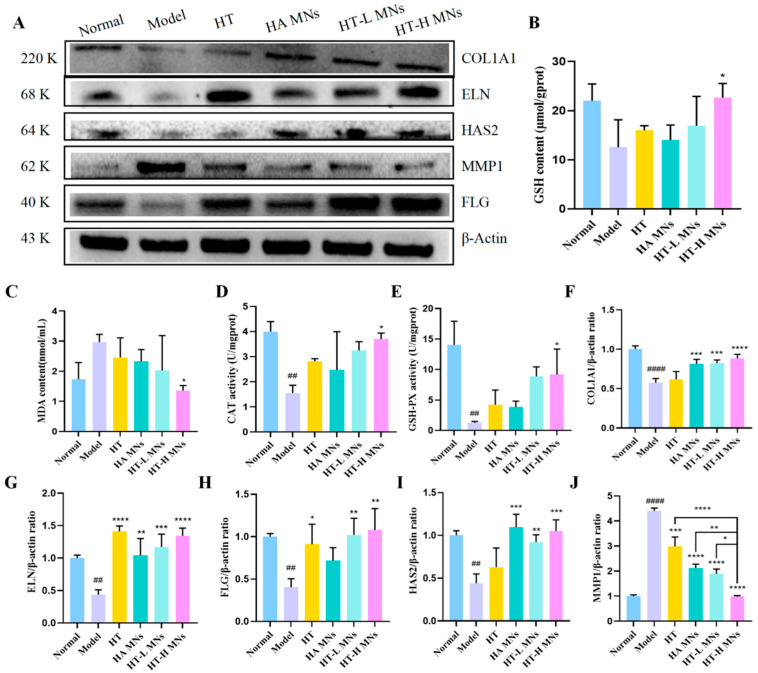
Regulation of Oxidative Stress and Safety Evaluation of HT MNs in Murine Skin Tissues. (**A**) Western blot analysis of oxidative stress-related protein expression. (**B**) Reduced GSH content (μmol/mg protein). (**C**) MDA content (nmol/mg protein). (**D**) CAT activity (U/mg protein). (**E**) GSH-PX activity (U/mg protein). (**F**) Relative expression of COL1A1 protein. (**G**) Relative expression of ELN protein. (**H**) Relative expression of FLG protein. (**I**) Relative expression of HAS2 protein. (**J**) Relative expression of MMP1 protein. Data points represent mean ± SD (n = 3 per group * *p* < 0.05, ** *p* < 0.01, *** *p* < 0.001, **** *p* < 0.0001, ## *p* < 0.01, #### *p* < 0.0001).

In stark contrast, the HT MNs treatment group, particularly the HT-H MNs group, demonstrated significant advantages in improving skin antioxidant status. This intervention markedly reduced skin MDA content, effectively elevated endogenous antioxidant GSH levels, and significantly enhanced CAT and GSH-Px enzyme activities. The positive changes in these biochemical indicators were highly consistent with the substantial improvement in skin appearance scores, collectively confirming that the HT MNs system can effectively ameliorate photoaging phenotypes by enhancing skin antioxidant defense capacity. Moreover, the effects exhibited a significant dose-dependent relationship, with the high-dose group showing the most pronounced outcomes (Figure 4B–E).

To investigate the molecular mechanisms underlying HT MNs-mediated improvement of skin photoaging, we employed Western blotting to assess the expression levels of key proteins involved in ECM synthesis and degradation, including COL1A1, ELN, HAS2, MMP1, and FLG. Compared to the normal control group, the model control group (UV-irradiated) exhibited an upward trend in MMP1 expression (1.25-fold, *p* > 0.05), a matrix-degrading enzyme. Concurrently, the expression of several critical structural and functional proteins was significantly suppressed, including FLG (0.40-fold, *p* < 0.01), ELN (0.44-fold, *p* < 0.01), HAS2 (0.44-fold, *p* < 0.01), and COL1A1 (0.58-fold, *p* < 0.0001), consistent with the ECM degradation and functional impairment observed in photoaged skin.

However, HT-H MNs intervention effectively reversed these abnormal protein expression profiles. Compared to the model control group, the HT-H MNs group significantly increased the protein expression of COL1A1 (1.54-fold, *p* < 0.0001), FLG (2.68-fold, *p* < 0.01), ELN (3.08-fold, *p* < 0.0001), and HAS2 (2.38-fold, *p* < 0.001), promoting ECM synthesis and repair. Simultaneously, this treatment markedly downregulated MMP1 expression (0.29-fold, *p* < 0.0001), effectively inhibiting excessive degradation of key ECM components such as collagen (Figure 4A,F–J). These molecular findings provide robust evidence that HT MNs repair photoaged skin by regulating ECM homeostasis.

### 2.12. Biocompatibility and Safety Evaluation of HT MNs in Mice

**Figure 5:**
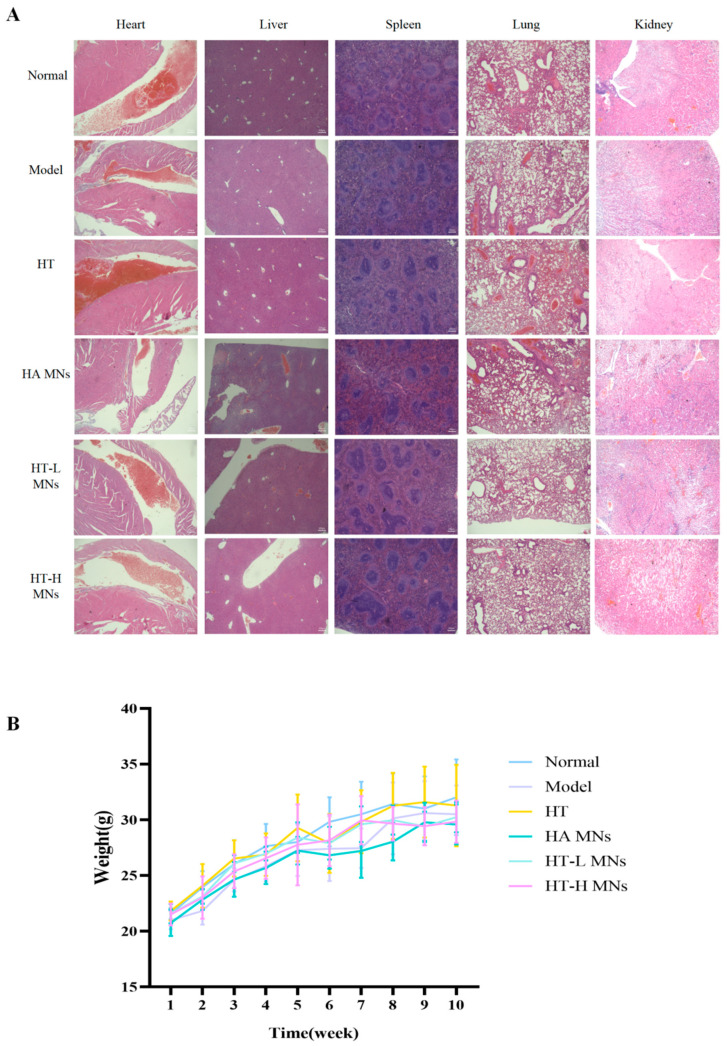
Safety evaluation of treatment in murine model. (**A**) H&E-stained sections of major organs (heart, liver, spleen, lung, kidney) collected at experimental endpoint (scale bar = 200 μm). All tissues exhibit normal histological architecture without evidence of inflammatory infiltration, necrosis, or fibrosis. (**B**) Body weight monitoring of ICR mice (n = 5/group) over 28-day treatment period. No significant differences in weight gain trajectories were observed between treated and vehicle control groups (two-way ANOVA, *p* > 0.05), indicating absence of treatment-related systemic toxicity.

Analysis of body weight trajectories and H&E staining of major organs revealed that dietary intake and metabolic parameters remained unaffected throughout the study period, indicating no adverse effects on systemic physiology. Specifically, HT MNs administration did not induce histopathological alterations in key immune-related organs, including the heart, liver, spleen, lung, and kidney. H&E-stained tissue sections demonstrated intact architectural integrity, with no evidence of inflammatory infiltrates, cellular degeneration, or fibrotic changes (Figure 5A,B). These findings collectively confirm the biocompatibility of HT MNs and their safety profile in preclinical models.

## 3. Discussion

The stratum corneum presents a formidable barrier for topical anti-photoaging agents, often leading to suboptimal bioavailability. MNs have emerged as a promising transdermal platform to overcome this limitation by creating transient microchannels that facilitate direct drug delivery to the dermal layer, where key photoaging processes occur [41]. This study developed a HT-loaded, HA-based soluble MN system (HT MNs), leveraging the dual benefits of mechanical penetration and the bioactive properties of both HT and HA.

First, the successful fabrication and robust mechanical properties of our HT MNs are crucial for effective transdermal delivery. The optimized formulation exhibited sufficient strength to penetrate murine skin (>200 μm), which is consistent with the performance metrics reported for other soluble polymeric MNs designed for dermatological applications [43]. The rapid self-recovery of the skin after MN removal underscores the minimally invasive nature of this approach, aligning with findings that MN-induced microchannels close within minutes to hours, minimizing infection risk [45]. Efficient HT delivery via this platform was therefore anticipated and subsequently confirmed by our pharmacological outcomes.

Our in vitro findings demonstrate that HT-MNs effectively protect human skin fibroblasts from UVA-induced photoaging. The significant reduction in intracellular ROS levels aligns with the well-documented potent antioxidant capacity of HT, which is known to scavenge free radicals and enhance endogenous antioxidant defense systems [46]. Furthermore, the suppression of SA-β-gal activity suggests an anti-senescence effect. This can be mechanistically linked to HT’s reported ability to modulate sirtuin pathways and mitigate DNA damage response, thereby delaying stress-induced premature senescence [47]. Importantly, the MN delivery format appeared to enhance these protective effects compared to free HT, likely by ensuring a higher localized concentration of HT directly within the target dermal fibroblasts, overcoming the penetration barrier that often limits topical antioxidants [48].

The superior in vivo efficacy of HT MNs was conclusively demonstrated in our UV-irradiated mouse model. The restoration of skin hydration and elasticity, along with the increased collagen density observed in Masson’s staining, directly correlates with the repair of dermal structure. Western blot analysis provided mechanistic insight: the upregulation of COL1A1 and ELN, coupled with the downregulation of MMP-1, indicates that HT MNs positively remodel the ECM. This regulatory pattern on collagen metabolism is consistent with studies showing that other antioxidant compounds can inhibit UV-activated AP-1 and NF-κB signaling pathways, which are upstream regulators of MMPs and procollagen synthesis [48]. The potent antioxidant activity of HT-MNs, evidenced by decreased MDA and elevated GSH/CAT/GSH-Px, likely underlies this ECM regulation, as oxidative stress is a primary driver of MMP induction and collagen degradation [49,50].

Compared to conventional topical HT or vitamin C, the HT MNs system offers a synergistic advantage. HA in the needle matrix not only provides structural integrity but may also contribute to skin hydration and wound healing [51,52,53], potentially creating a favorable microenvironment for HT activity. This multi-component synergy represents a significant advancement over single-agent topical treatments.

Despite these promising results, our study has limitations that point to future research directions. The precise intracellular signaling pathways (e.g., NF-κB, MAPK, Nrf2) through which HT MNs exert their effects warrant further investigation using specific inhibitors or genetic approaches. Furthermore, optimizing the formulation for sustained release to prolong therapeutic effect and scaling up drug loading for human applications are critical translational steps [54].

In conclusion, this study provides compelling evidence that HT-loaded soluble MNs represent a novel and effective strategy against skin photoaging. By ensuring efficient dermal delivery, HT MNs potentiate the known antioxidant and anti-senescence properties of HT, leading to significant improvements in skin structure, ECM homeostasis, and oxidative stress markers in vivo [55,56,57]. These findings establish a strong foundation for the development of MN-based combinatory therapies in dermatology and aesthetic medicine.

## 4. Materials and Methods

### 4.1. Materials

HA (Mw = 200 kDa) was obtained from Shandong Focus Biotechnology Co., Ltd. (Jinan, China). HT (purity ≥ 98%) was purchased from Nanjing Hegu Life Biotechnology Co., Ltd. (Nanjing, China). PVP K90 (analytical grade) was purchased from Wuxi Yatai United Chemical Co., Ltd. (Wuxi, China). The PDMS used for the MN mold was sourced from Henan Weina Bengteng Biotechnology Co., Ltd. (Zhengzhou, China). All other reagents are of analytical grade.

HSFs (HTX2132) were purchased from Shenzhen Haodi Huatuo Biotechnology Co., Ltd. (Shenzhen, China). Maintain the cells in a modified medium (DMEM, Gibco, Grand lsland, NY, USA) supplemented with 10% fetal bovine serum (FBS, Gibco, Grand lsland, NY, USA) and 1% penicillin/streptomycin (Gibco, Grand lsland, NY, USA) at a temperature of 37 °C and humidity of 5% CO_2_ atmosphere. Female ICR mice (6–8 weeks old, 18–22 g) were obtained from Shandong Pengyue Experimental Animal Center (Jinan, China). Keeping animals under Specific Pathogen Free (SPF) conditions (22 ± 2 °C, 50 ± 10% humidity, 12 h of light/dark cycle) allows for free access to food and water. All programs have been approved by the Animal Care and Use Committee of the New Drug Evaluation Center of Shandong Academy of Pharmaceutical Sciences (Approval Number: care-2025003).

### 4.2. Screening of Matrix Materials and Preparation Processes for HT MNs

To screen for the optimal preparation parameters of HT MNs, the MNs prepared were evaluated based on factors such as the ease of film release, array integrity, bubble volume, needle type, and needle tip hardness. After the effects of solvent type, needle tip material composition, backing material ratio, vacuum time, and drying method on MN performance were systematically examined, the most suitable preparation materials and processes were selected (Tables S2).

### 4.3. Preparation of HA MNs Loaded with HT

A 10% (*w*/*v*) HA solution was prepared by dissolving 5 g of 200 kDa HA in 50 mL deionized water with magnetic stirring at 60 °C until complete dissolution. HT was then added at 10% (*w*/*w*) relative to HA, and the mixture was sonicated for 30 min for uniform mixing. Next, 2 mL of the HT/HA mixture was transferred into a 10 × 10 PDMS MN mold (needle height: 548.5 μm; base diameter: 344.4 μm). The mold was vacuumed at −0.7 MPa for 15 min to remove bubbles and ensure full injection of the solution. Afterwards, 150 μL of a 10% (*w*/*v*) PVP K90 solution was cast onto the mold surface to form a backing layer. The mold was dried at 37 °C for 12 h to evaporate the solvent and solidify the layer. Finally, the MN array was peeled off and stored in a desiccator at room temperature until use.

### 4.4. Visualization of MNs

The surface morphology of the prepared MNs was characterized by means of visual inspection, camera photography, and SEM. For SEM analysis, the MNs were first coated with a thin layer of carbon. Subsequently, they were visualized using an FEI Nova NanoSEM (Eindhoven, The Netherlands) operated at a voltage of 15.0 kV.

### 4.5. Mechanical Properties of MNs

Compression displacement tests were conducted on HT MNs using a universal testing machine. A complete MN tip was positioned facing upwards and fixed perpendicular to the testing platform surface. An axial force, orthogonal to the array axis, was applied and incrementally increased. Upon contact between the mobile sensor and MNs, the displacement of the MN under the applied force was recorded, and a mechanical displacement curve was plotted for subsequent analysis of the MN’s mechanical strength.

### 4.6. Skin Penetration Efficiency and Recovery Performance of MNs

After inserting MNs into mouse skin, the skin was stained with a 0.25% (*v*/*v*) trypan blue (TB) staining solution for 5 min. The skin surface was then washed with PBS buffer to eliminate excess dye [44]. The number of holes stained with TB was observed and captured using a camera, and the skin penetration efficiency (PE) of the MNs was calculated using the formula: PE = (number of stained holes/number of pinholes). Meanwhile, the various stages of skin recovery were documented, and an assessment was made to determine whether the damage induced by the MNs on the skin was reversible, as well as to evaluate the recovery of small pores on the skin over a specified period [44,45].

### 4.7. Determination of Drug Content of MNs

High performance liquid chromatography (HPLC) method was used to determine the drug content in MNs. By comparing the retention time and peak area of the standard sample, the concentration of HT in the test sample was calculated, and then the drug content of HT MNs was determined.

### 4.8. Cell Culture and In Vitro Photoaging Model Construction

HSFs were seeded into 6-well plates at a density of 2 × 10^5^ cells per well and cultured in DMEM supplemented with 10% FBS and 1% penicillin/streptomycin at 37 °C in a humidified 5% CO_2_ incubator. Upon reaching 70–80% confluence, the cells were exposed to UVA radiation using a UV crosslinker (Scientz03-II, Shanghai, China).

Subsequently, the HSFs were inoculated into a 96-well plate, divided into six groups with three replicates each, and irradiated with UVA at doses of 0, 5, 10, 15, 20, and 25 mJ/cm^2^, respectively. After 24 h of cultivation, 100 µL of culture medium and 10 µL of CCK-8 solution (Beyotime, Shanghai, China) were added to each well, followed by incubation in the incubator for 1 h. Cell viability was assessed using the CCK-8 assay, and absorbance was measured at 450 nm with an INFINITE M200PRO Full-Wavelength Microplate Reader (TECAN, Shanghai, China). Cell-free culture medium served as the blank control. The cell survival rate was calculated using the formula: Cell survival rate (%) = [(control group − experimental group)/(control group − blank group)] × 100%, to determine the optimal UV irradiation dose.

### 4.9. Cell Administration and Vitality Testing

HSFs were seeded into a 24-well plate and randomly assigned to four experimental groups: (A) Normal group: Cells were cultured under standard conditions without UV exposure or MN intervention, serving as the baseline control. (B) Model group: Cells were exposed to UV irradiation without MN treatment to mimic pathological conditions. (C) HA MNs group: Blank HA MNs (9 mg/mL) were used as negative controls to evaluate non-specific effects. (D) HT MNs group: Cells were treated with UVA and a MN array loaded with HT at a concentration of 9 mg/mL, applied directly to the cell monolayers. As described previously, Cell viability was assessed via CCK-8 assay according to the standard procedure. The measured absorbance at 450 nm was used to calculate the cell survival rate, which facilitated the selection of the optimal UV irradiation dose for follow-up studies.

### 4.10. Cellular Senescence Assay (SA-β-Gal Staining)

Cells were inoculated into 24-well plates at a density of 5 × 10^4^ cells per well and randomly assigned to four experimental groups (n = 3 wells per group) for subsequent (A) Normal group, (B) Model group, (C) HA MNs group, and (D) HT MNs group. After 24 h of adherent culture, the medium was removed. Except for the normal control group, 200 μL of PBS was added to each well of the remaining three groups, followed by irradiation using a UVA irradiator (irradiation dose: 25 mJ/cm^2^). After irradiation, the PBS was discarded, complete medium was replenished, and the cells were further incubated at 37 °C in a humidified 5% CO_2_ atmosphere for 4 h. Subsequently, the model group received no additional treatment, while the HA and HT groups were treated with medium containing hyaluronic acid (9 mg/mL) or hydroxytyrosol (9 mg/mL), respectively. All groups were then cultured for an additional 24 h. Senescent cells were identified using a SA-β-gal staining kit (Beyotime, Shanghai, China), adhering strictly to the manufacturer’s guidelines. Briefly, after rinsing with PBS, cells in each well were fixed with 1 mL of staining fixative solution at room temperature for 15 min. This was followed by three rounds of PBS washing, with each wash lasting 3 min. Subsequently, 1 mL of freshly prepared staining solution was added to each well. The plates were then sealed with parafilm to prevent evaporation and incubated overnight at 37 °C in a non-CO_2_ incubator. Following incubation, senescent cells, which appeared as dark blue-stained cells, were observed under a bright-field microscope (Nikon ECLIPSE Ti-S Dual-Port Inverted Microscope, Tokyo, Japan). To quantify the β-galactosidase-positive area, the mean optical density (MOD) of three randomly selected fields per well was analyzed using ImageJ software (Image Pro Plus 6.0) [51].

### 4.11. ROS Detection

The procedures for cell seeding, grouping, and treatment were conducted in accordance with the description provided in Section 4.10 above. Intracellular ROS levels were measured using a Reactive Oxygen Species Assay Kit (Beyotime, Shanghai, China) [45]. Prior to detection, the DCFH-DA probe was diluted 1:1000 in PBS to prepare a working solution with a final concentration of 10 μM. After gentle washing with PBS, cells were incubated with 500 μL of DCFH-DA working solution per well at 37 °C in the dark for 20 min. Following incubation, the cells were thoroughly washed three times with PBS to completely remove any extracellular probe. Fluorescence images were then captured using a fluorescence microscope (EVOS FL, Thermo Fisher, Waltham, MA, USA) at excitation/emission wavelengths of 488/525 nm. Three random fields per well were selected, and the mean fluorescence intensity (MFI) was analyzed and calculated using ImageJ software (National Institutes of Health, Bethesda, MD, USA) [53].

### 4.12. Pharmacodynamic Study In Vivo

Female ICR mice, aged 6–8 weeks and weighing 18–22 g, were acclimatized for one week prior to experimentation. The dorsal fur of each mouse was removed via shaving and chemical depilation to ensure uniform UV exposure. The minimal erythema dose (MED) was predefined as the lowest cumulative radiation dose of UVA and UVB that resulted in discernible skin erythema. The MED was empirically determined to be a combined dose of 90 mJ/cm^2^ UVA and 60 mJ/cm^2^ UVB. Photoaging was induced using a SS-01 and SH1 UV Phototherapy Apparatus. The irradiation protocol consisted of UVA and UVB exposure over a 4-week period, with the weekly dose progressively increased from 1 MED to 4 MED. This regimen yielded a total cumulative dose of 10 MED, which is biologically equivalent to approximately four weeks of natural solar radiation. Upon completion of the 4-week irradiation schedule, the mice were randomly assigned to one of six groups (n = 5 per group) using a random number generator to ensure unbiased group allocation. There was no UV radiation/treatment in the normal group; The model group was treated with UV radiation; simple HA MNs Group: A was treated with ultraviolet radiation and local HA solution (10 mg/mL); HT-L MNs Group: A was treated with ultraviolet radiation + HT loaded MNs (5 mg/mL); HT-H MNs Group: A was treated with ultraviolet radiation + HT loaded MNs (10 mg/mL). HT group was treated with UV radiation + local HT solution (10 mg/mL). The array (15 × 15 needles, height = 548.5 ± 25 μm) was applied to the UV irradiated skin using a spring-loaded applicator (force = 0.5 N/needle). The treatment was conducted once every two weeks for 4 weeks. In the topical HT application group, 10% (*w*/*v*) ht solution (100 μL/cm^2^) was applied to the back skin every day for 4 weeks. A digital camera (Nikon D3100, Tokyo, Japan) was used to capture the skin conditions of mice at different time points.

### 4.13. Apparent Evaluation of Mouse Skin

The skin appearance image was captured using a mobile phone. Based on the scheme previously described by Zhang [57] and others, the light damage was evaluated by visual score, which was performed by two observers who were not aware of the grouping (Table 1). And adjust the temperature and humidity of the laboratory to the appropriate level, which is generally 22 ± 2 °C, and the relative humidity is 40%~70%. The moisture and elasticity of mouse skin were evaluated using a skin tester. After cleaning and disinfecting the test probe, gently place it on the surface of the depilated skin of mice, start the instrument, measure the skin moisture and elasticity according to the set procedure, and record the measurement data.

Table 1 provides a detailed listing of the grading criteria for skin appearance wrinkles, aiming to offer a clear and actionable reference framework for accurately assessing the condition of skin wrinkles.

### 4.14. Histological Examination

Following the conclusion of the treatment, the mice were euthanized via CO_2_ inhalation. Specifically, the mice were placed in a suitable chamber, and a controlled flow of CO_2_ gas was gradually introduced to displace the air in the chamber, ensuring a humane and painless death for the animals. Subsequently, the carcasses of the mice were uniformly handled by the New Drug Evaluation Center for proper disposal. After treatment, the skin of the animals was taken and fixed with 4% paraformaldehyde. The tissues were decalcified with EDTA solution. After decalcification, paraffin embedded sections were made. H&E and Masson were used for staining. The stained sections were observed under a microscope and photographed at three randomly selected positions. The images were processed by image analysis software ImageJ to observe the skin tissue morphology and collagen content, and the skin aging was scored.

### 4.15. Determination of Indicators Related to Oxidative Stress

Oxidative stress is a core and complex concept in biology and medicine. It describes a state of imbalance between the oxidative and antioxidant systems within cells or organisms, where the generation of ROS exceeds the body’s capacity for clearance, leading to potential molecular damage and functional disorders [58]. Organisms have evolved a sophisticated antioxidant defense system to maintain redox balance [59]. This system includes enzymatic antioxidant systems, such as superoxide dismutase SOD, CAT, and GSH-Px, which efficiently catalyze the removal of specific ROS. It also comprises non-enzymatic antioxidant molecules, including GSH, vitamin C, and flavonoids, which function by directly reacting with ROS or regenerating other antioxidants [50,51].

Pre-treatment of cell samples: After trypsin digestion, centrifuge the cell suspension at 1000 r/min for 10 min, discard the supernatant, and retain the cell pellet. Wash the pellet 1–2 times with an isotonic buffer solution, centrifuging again at 1000 r/min for 10 min each time, discarding the supernatant, and retaining the cell pellet. Add 0.2–0.3 mL of homogenization medium (recommended: 0.1 mol/L PBS or physiological saline with a pH of 7–7.4) to the cell pellet. Gently mix the cell solution after adding the homogenization medium to ensure uniformity, then take a small amount for cell counting. Lyse the cells using Triton X-100 lysis buffer for 30 min.

MDA, as one of the key end products of lipid peroxidation, serves as a core biomarker for evaluating oxidative stress levels in organisms or food products [60]. MDA content was determined using an MDA content assay kit (A007-1-1, Nanjing Jiancheng Bioengineering Institute, Nanjing, China) via the thiobarbituric acid (TBA) method. Briefly, the cell lysis supernatant was mixed with TBA working solution, heated in a 95 °C water bath for 40 min, cooled, and then centrifuged. The supernatant was collected, and absorbance was measured at 532 nm. MDA content was calculated using a standard curve and expressed as nanomoles per milligram of tissue protein (nmol/mg prot).

GSH is the most abundant non-protein thiol compound within cells and plays a pivotal role in maintaining cellular redox homeostasis [61]. Accurate and efficient detection of GSH content in cells or biological samples is crucial for assessing cellular health status, studying oxidative stress, screening drugs, and diagnosing diseases [61]. The microplate method, particularly those utilizing 96-well or 384-well plates, has become the mainstream approach for GSH quantification due to its high throughput, ease of operation, minimal sample consumption, and suitability for automation [62]. GSH content was measured using a reduced GSH content assay kit (A006-2-1, Nanjing Jiancheng Bioengineering Institute, Nanjing, China) via the microplate method. This method involves the reaction of GSH with 5,5′-dithiobis-(2-nitrobenzoic acid) (DTNB) to produce a yellow product, 5-thio-2-nitrobenzoic acid (TNB), whose absorbance was measured at 412 nm. GSH content was calculated using a standard curve and expressed as micrograms per milligram of tissue protein (μg/mg prot).

CAT activity was determined using a CAT assay kit (A007-1-1, Nanjing Jiancheng Bioengineering Institute, Nanjing, China) via the ammonium molybdate method. After collecting the cells, add 0.3 mL of physiological saline (or PBS) to each sample (the cell count should preferably not be less than 10^6^ cells). Ultrasonically disrupt the cells in an ice-water bath (power: 200–300 W, run for 5 s, pause for 15 s, repeat 3–5 times). Centrifuge at 4000 r/min for 10 min, collect the supernatant, and measure the protein concentration. Add the working solution according to the instructions and reflect CAT activity by measuring the rate of decrease in absorbance of H_2_O_2_ at 240 nm [63,64].

GSH-Px activity was measured using a GSH-Px assay kit (A005-1, Nanjing Jiancheng Bioengineering Institute, Nanjing, China) via a colorimetric method. The principle is that GSH-Px catalyzes the reaction between hydrogen peroxide (H_2_O_2_) and reduced GSH to produce water (H_2_O) and oxidized glutathione (GSSG). The activity of glutathione peroxidase can be represented by the rate of this enzymatic reaction. By measuring the consumption of reduced glutathione during the enzymatic reaction, the enzyme activity can be determined [65]. Take the cell supernatant, add the working solution, and mix well. Centrifuge at 3500–4000 r/min for 10 min, take 1 mL of the supernatant for the color reaction, add the chromogenic solution, mix well, and let it stand at room temperature for 15 min. Measure the optical density (OD) value of each tube at 412 nm using a 1 cm light path cuvette, with distilled water as the blank.

Western Blotting Analysis: We collected the cells and lysed them with radio immunoprecipitation assay (RIPA) lysis buffer plus protease inhibitors. Cells were lysed on ice for 40 min, followed by centrifugation at 12,000 rpm for 15 min at 4 °C. We further collected the supernatant and measured the protein concentration using the Bicinchoninic acid (BCA) protein kit. We electrophoresed the extracted cell lysates on 10% sodium dodecyl sulfate-polyacrylamide gel electrophoresis (SDS-PAGE) separating gel and transferred the proteins to poly(vinylidene fluoride) (PVDF) membranes (Immobi-lon-P; Millipore, Billerica, MA, USA). Following 1 h blocking with nonfat milk, the membranes were incubated with primary antibody overnight at 4 °C. The horseradish peroxidase-conjugated secondary antibody was diluted 1:2000 and incubated for 1 h at room temperature. On the following day, wash the membrane with TBST, and then incubate it with the corresponding species-specific horseradish peroxidase (HRP)-conjugated secondary antibody at room temperature for 1 h. The membranes were detected by using enhanced chemiluminescence. Each experiment was repeated three times, and all protein expressions were normalized to internal controls. Develop the samples using enhanced chemiluminescence (ECL) substrate on a chemiluminescence imaging system. Analyze the gray values of the target bands using ImageJ software, and calculate the relative expression levels of the protein of interest by using β-actin as an internal reference [66].

### 4.16. In Vivo Safety Evaluation

To objectively assess experimental safety, mouse body weight was monitored throughout the study. Following the experimental endpoint, major organs (heart, liver, spleen, lung, and kidney) were harvested from euthanized mice and immediately fixed in 4% (*w*/*v*) paraformaldehyde (PFA) for 24 h at 4 °C. The fixed tissues were then embedded in paraffin, sectioned at 5 μm thickness, and subjected to H&E staining for histological analysis. This approach enabled the detection of histopathological alterations and assessment of drug-induced toxicity in target organs.

### 4.17. Statistical Analysis

In this study, each experimental group consisted of at least three biological replicates. Data are presented as mean ± standard deviation (SD). Statistical analyses were performed using Microsoft Excel (version 2019 or later) for preliminary calculations and IBM SPSS Statistics (version 26.0) for advanced statistical modeling. For comparisons between two groups, unpaired Student’s *t*-test was applied (with Welch’s correction for unequal variances). Multiple-group comparisons were conducted using one-way ANOVA followed by Tukey’s post hoc test. All statistical analyses and graph generation were performed with GraphPad Prism (version 8.0). Statistical significance thresholds were set at * *p* < 0.05, ** *p* < 0.01, *** *p* < 0.001, and **** *p* < 0.0001.

## Figures and Tables

**Table 1:** Grading criteria for skin appearance wrinkles.

Score	Grading Criteria
0	No obvious wrinkles; skin is smooth and firm
1	Slight wrinkles, only visible during facial expressions; good skin elasticity.
2	Moderate wrinkles, visible at rest; average skin elasticity
3	Severe wrinkles, prominently visible at rest; skin is lax with poor elasticity.

## Data Availability

The original contributions presented in this study are included in the article/Supplementary Material. Further inquiries can be directed to the corresponding authors.

## Supplementary Materials

The following supporting information can be downloaded at https://www.mdpi.com/article/10.3390/ijms27021005/s1.

## Abbreviations

The following abbreviations are used in this manuscript:

CAT	catalase
cck-8	cell counting kit-8
COL1A1	collagen type I alpha 1
DCF	2′,7′-dichlorofluorescein
DCFH	2′,7′-dichlorodihydrofluorescein
DCFH-DA	2′,7′-dichlorodihydrofluorescein diacetate
ECL	enhanced chemiluminescence
PDMS	poly-dimethylsiloxane
ELN	elastin
FBS	fetal bovine serum
FLG	filaggrin
GSH	glutathione
GSH-Px	glutathione peroxidase
H&E	hematoxylin-eosin
HA	hyaluronic acid
HPLC	high performance liquid chromatography
HSF	human skin fibroblasts
HT	hydroxytyrosol
IC50	half maximal inhibitory concentration
MDA	malondialdehyde
MED	minimal erythemal dose
MMPs	matrix metalloproteinases
MMP1	matrix metalloproteinase 1
MNs	microneedles
NADPH	nicotinamide adenine dinucleotide phosphate
NF-κB	nuclear factor kappa B
PBS	phosphate-buffered saline
PDMS	polydimethylsiloxane
PVP	polyvinyl pyrrolidone
ROS	reactive oxygen species
SA-β-Gal	senescence-associated β-galactosidase
SDS-PAGE	sodium dodecyl sulfate-polyacrylamide gel electrophoresis
SEM	scanning electron microscope
UV	ultraviolet
UVA	ultraviolet A
UVB	ultraviolet B
