# Supplementary material for: Anti-Photoaging Effect of Soluble Microneedles Loaded with Hydroxytyrosol
Source: Int J Mol Sci. 2026 Jan 20;27(2):1005. doi: 10.3390/ijms27021005 (PMC12841782; doi:10.3390/ijms27021005)
Supplement: Supplementary file 1 [file ijms-27-01005-s001.zip › ijms-4065485-supplementary.pdf]

Supplementary Materials of

Anti-photoaging effect of soluble microneedles loaded with hydroxytyrosol

Jie Wang[a,b,#], Gaofei Zhu[a,b,#], Mengke Han[a,b], Xinyu Hou[a,b], Yishu Wang[b,c], Xiuhua Zhang[b], Jinhua Zhang[b], Huarong Shao[a,b,*], Fei Liu[a,b,*]

[a] *School of Pharmacy, Shandong University of Traditional Chinese Medicine, Jinan, Shandong, China*

[b] *Engineering Research Center for Sugar and Sugar Complex, National-Local Joint Engineering Laboratory of Polysaccharide Drugs, Key Laboratory of Carbohydrate and Glycoconjugate Drugs, Shandong Academy of Pharmaceutical Science, Jinan, Shandong, China*

[c] *Key Laboratory of Brain, Cognition and Education Sciences, Ministry of Education, China; Institute for Brain Research and Rehabilitation, South China Normal University, Guangzhou , Guangdong ,China.*

E-Mail address: wangjie91013@q63.com(J.Wang);shaohuarong@sdaps.cn (H. Shao);liufei@sdaps.cn (F. Liu)

Table S1. Scores of formability, mechanical strength and bubble volume of each MN. This table can be used to compare and evaluate the performance of different MNs - related experimental conditions.No.1 (HT MNs) has relatively high scores across most indicators, resulting in a total score of 13, while many other samples have low or zero scores, suggesting significant issues in multiple areas.

Number	Single Indicator Score					Total Score
	Difficulty of film removal	Array integrity	Bubble quantity	Needle type	Needle tip hardness	
**1 (HT MNs)**	1	3	3	3	3	13
**2 (Ethanol solvent)**	0	0	0	0	0	0
**3 (high concentration HT)**	1	2	2	2	0	7
**4 (high molecular weight HA)**	0	0	0	0	0	0
**5 (high concentration HA)**	0	0	0	0	0	0
**6 (CMC-Na)**	0	0	0	0	0	0
**7 (without PVPK90)**	1	3	3	3	0	10
**8 (high concentration PVPK90)**	0	1	1	1	3	6
**9 (short vacuum time)**	1	0	0	0	0	1
**10 (natural air drying)**	0	1	1	1	0	3

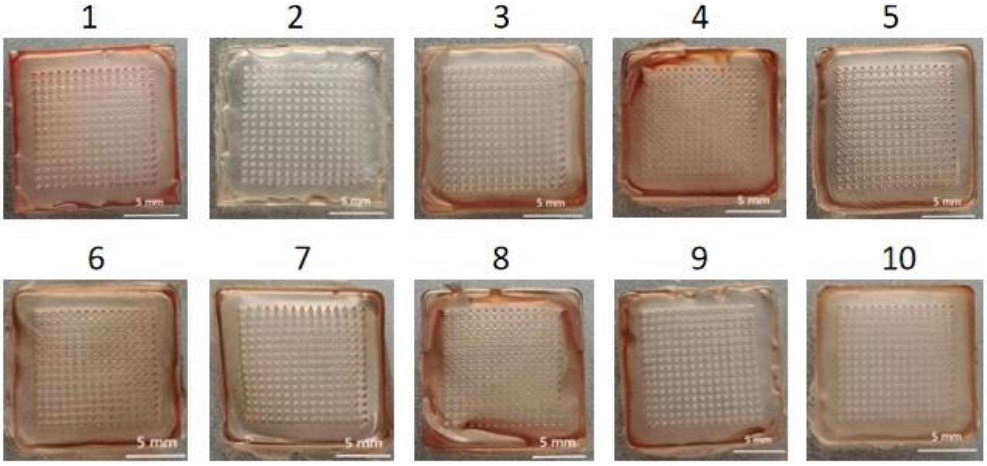

Figure S1. Photographs of each MN.Scale bar=5 mm. The items numbered 1 - 10 in the figure correspond to the ten samples in Table S1 - 1 respectively.（For example，picture 1 is the image of Sample 1, which is HT MNs.）

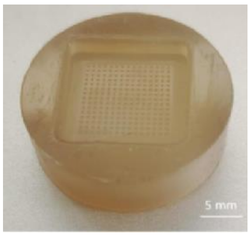

Figure S2. MNs mold image. Scale bar=5 mm.

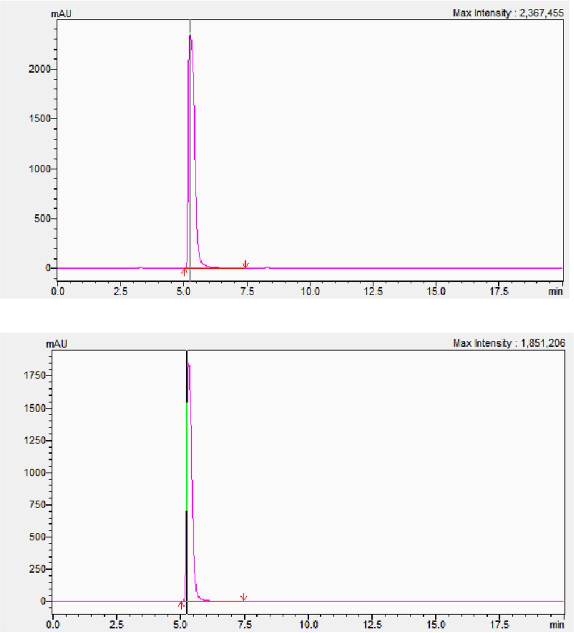

Figure S3. Identification Diagram of Drug Loading Capacity of HT MNs. The chromatography column is AQ-C18 (4.6×150 mm, 5 μm). The detector wavelength is set at 276 nm, with a flow rate of 1.0 mL/min, an injection volume of 10 μL, and a column temperature of 35 ℃. The run time is 20 min under isocratic elution. For reagents and solutions, glacial acetic acid (analytical grade) and methanol (chromatographic grade) are used. The mobile phase is a ratio of 80:20 for mobile phase A to B. Mobile phase A is prepared by adding 1.0 mL of acetic acid to 1000 mL of water, filtered through a 0.22 μm membrane, while mobile phase B is methanol. The diluent is the mobile phase.

Table S2A Matrix materials and preparation processes for different MNs. This table details the matrix materials and preparation methods for various MN types, providing valuable insights into the material - selection and fabrication aspects of microneedle technology.

Number	Solvent	matrix solution	backing layer	vacuum time	drying temperature
**1(HT MNs)**	Deionized water	10%HT+5% 200 kDaHA	10%PVPK90	15 min	37 °C
**2 (Ethanol solvent)**	50% ethanol	10%HT+5% 200 kDaHA	10%PVPK90	15 min	37 °C
**3 (no HT)**	deionized water	5% 200 kDaHA	10%PVPK90	15 min	37 °C
**4 (high concentration HT)**	deionized water	20%HT+5% 200 kDaHA	10%PVPK90	15 min	37 °C
**5 (high molecular weight HA)**	deionized water	10%HT+5% 1200 kDaHA	10%PVPK90	15 min	37 °C
**6 (high concentration HA)**	deionized water	10%HT+10% 200 kDaHA	10%PVPK90	15 min	37 °C
**7(CMC-Na)**	deionized water	10%HT+5% 200 kDaHA	CMC-Na	15 min	37 °C
**8 (without PVPK90)**	deionized water	10%HT+5% 200 kDaHA	HA	15 min	37 °C
**9 (high concentration PVPK90)**	deionized water	10%HT+5% 200 kDaHA	20%PVPK90	15 min	37 °C
**10 (short vacuum time)**	deionized water	10%HT+5% 200 kDaHA	10%PVPK90	5 min	37 °C
**11 (naturally air dried)**	deionized water	10%HT+5% 200 kDaHA	10%PVPK90	15 min	25 °C

Table S2B MN Product Quality Scoring Criteria. This table outlines a scoring system for evaluating different aspects of microneedles (MNs). It consists of five scoring items: Demolding Ease, Array Integrity, Bubble Content, Needle Shape. For each item, detailed scoring criteria are provided, ranging from 0 (indicating severe issues that may render the MNs unusable or significantly impair their functionality) to 2 or 3 (representing optimal conditions with no or minimal defects). The criteria cover a wide range of potential problems, such as difficulties in demolding, damage to the MN array, presence of bubbles, and needle shape irregularities. This scoring system can be used as a standardized method for quality control and performance assessment of MNs during manufacturing and research processes.

Scoring Item	Scoring Criteria
**Demolding Ease**	0: Difficult to demold; requires strong pulling or tool assistance, with severe residue or large-area damage.<br>1:Requires slight force to demold; has minor residue or localized damage, but does not affect overall use.<br>2:Easy to demold; no residue or damage, completed in a single step.
**Array Integrity**	Large areas of the array are missing, fractured, or severely deformed; unusable.<br>1:More than 3 microneedles are damaged or the arrangement is significantly uneven; functionality is partially impaired.<br>2:1-2 minor defects or slight misalignment in the array; overall functionality is not significantly affected.<br>3:The array is complete, with no damage or fractures; arrangement is uniform and density meets design specifications.
**Bubble Content**	0:Bubbles are dense or located in critical areas (e.g., needle tips), causing a loose structure or functional failure.

	1:Numerous bubbles or presence of large bubbles, which may affect drug release or mechanical strength.<br>2:A few tiny bubbles present in non-critical areas (e.g., the base), with no impact on performance.<br>3:No visible bubbles; the microneedle structure is dense and uniform.
**Needle Shape**	0:Severe needle deformation (e.g., fracture, hooks); completely unusable.<br>1:Significant blunting or deformation (e.g., bending, flattening), with a notable decrease in penetration efficiency.<br>2:Slight blunting or minor shape deviation (e.g., slightly rounded tip), but does not affect penetration capability.<br>3: Sharp, regular shape; the tip is fully consistent with design requirements.
